# Isolated Follicle-Stimulating Hormone (FSH) Deficiency in Male Sex: A Case Report

**DOI:** 10.7759/cureus.100496

**Published:** 2025-12-31

**Authors:** Daniela M Soares, Jorge Diogo Silva, Ana Rita Soares, André Couto de Carvalho

**Affiliations:** 1 Endocrinology Department, Unidade Local de Saúde de Santo António, Porto, PRT; 2 Medical Department, Centro de Genética Médica Doutor Jacinto Magalhães (CGM), Unidade Local de Saúde de Santo António, Porto, PRT

**Keywords:** fsh deficiency, fsh-β mutations, gynecomastia, male hypogonadism, male infertility

## Abstract

Follicle-stimulating hormone (FSH) is a glycoprotein hormone produced in the anterior pituitary, essential in the regulation of gonadal functions. Isolated FSH deficiency (IFD) is a rare inherited disorder, usually caused by β-subunit alterations. In men, it is frequently detected during infertility evaluation, commonly associated with spermatogenesis impairment and testicular atrophy with normal testosterone levels.

We present the case of a 30-year-old male patient who was referred for breast pain and bilateral gynecomastia. Laboratory evaluation displayed primary hypothyroidism with positive thyroid autoantibodies and decreased FSH levels, with normal total testosterone and adequate luteinizing hormone (LH) levels. A gonadotropin-releasing hormone (GnRH) stimulation test demonstrated inadequate FSH response, highly suggestive of IFD. Genetic testing for *FSHB* mutations was negative, and whole exome sequencing revealed no recognized pathogenic variants. Semen analysis was postponed by the patient’s choice.

Although rare, IFD should be considered when evaluating patients with symptoms suggestive of hypogonadism. The overall prevalence of FSH-β mutations is unknown but probably underdiagnosed in male patients.

## Introduction

Follicle-stimulating hormone (FSH) is a glycoprotein hormone produced by the anterior pituitary in response to hypothalamic gonadotropin-releasing hormone (GnRH). It plays a key role in reproductive function, stimulating ovarian follicle maturation in women and inducing Sertoli cell proliferation and spermatogenesis in men [1]. FSH comprises two subunits: the α-subunit, shared with other pituitary hormones (e.g., thyroid-stimulating hormone (TSH) and luteinizing hormone (LH)), and the β-subunit, which grants FSH its specificity [2].

Isolated FSH deficiency (IFD) is a rare condition characterized by hypogonadism without impairment of other pituitary hormonal axes. It is caused by autosomal recessive (AR) pathogenic variants in the *FSHB *gene, located on chromosome 11p13, which encodes the FSH β-subunit. Nevertheless, several cases without a clear molecular diagnosis even after genetic testing have been described [3-6]. In men, it manifests with spermatogenesis impairment and testicular atrophy; evaluation of the gonadal axis is required and typically reveals normal testosterone and LH but with low FSH levels and poor or absent FSH response to GnRH administration [2,3]. This condition is commonly detected during fertility evaluation; however, its true prevalence remains unknown, as the medical literature is limited to sporadic case reports and lacks robust population-based prevalence data.

## Case presentation

A 30-year-old male patient, working as a professional horse rider, was referred to our Endocrinology clinic due to right breast pain and gynecomastia lasting six months. The patient denied galactorrhea, steroid use, libido changes, sexual dysfunction, facial hair changes, headache, visual impairment, nausea, or anosmia. Notably, he had a history of recurrent horse falls with occasional head injuries and frequent use of opioids and muscle relaxants. Family history was negative for fertility disorders or consanguinity. Physical examination revealed normal secondary sex characteristics and painless palpable right gynecomastia, without discharge or palpable axillary lymph nodes. Testicular examination revealed normal volume testicles (25 mL), with no palpable nodules. Breast ultrasound confirmed right-sided predominant gynecomastia without nodular lesions. First hormonal evaluation revealed decreased FSH levels (0.6 UI/L (1.5-12.4)) and mildly elevated TSH levels (Table 1).

**Table 1:** Laboratory findings FSH: follicle-stimulating hormone; HCG: human chorionic gonadotropin; IGF-1: insulin-like growth factor-1; LH: luteinizing hormone; LT4: levothyroxine; T3: triiodothyronine; T4: thyroxin; Tg: thyroglobulin; TPO: thyroid peroxidase; TSH: thyroid-stimulating hormone SI unit converter: cortisol (nmol/L) = (µg/dL) * 27.60; prolactin (mIU/L) = (ng/mL) * 21.20; free T4 (pmol/L) = (ng/dL) * 12.87; free T3 (pmol/L) = (pg/mL) * 1.54; total testosterone (nmol/L) = (ng/mL) * 3.47; estradiol (pmol/L) = (pg/mL) * 3.67; IGF-1 (nmol/L) = (ng/mL) * 0.13

	First evaluation	Six-month evaluation	Five months after LT4 initiation	Reference range
Cortisol	11.6	-	-	6.2-19.4 µg/dL
Prolactin	15.5	14.8	15.0	4.04-15.2 ng/mL
TSH	6.06	15.10	3.09	0.30-3.18 mUI/L
Free T4	1.18	0.87	1.42	1.01-1.65 ng/dL
Free T3	3.56	3.09	3.88	2.66-4.33 pg/mL
Anti-TPO antibody	-	141	-	Negative < 34 UI/mL
Anti-Tg antibody	-	774	-	Negative < 115 UI/mL
FSH	0.6	0.6	0.6	1.5-12.4 UI/L
LH	4.4	5.2	4.1	1.7-8.6 UI/L
α-subunit	-	0.11	-	0.00-0.80 UI/L
Total testosterone	3.52	3.37	4.39	2.8-8.0 ng/mL
Estradiol (E2)	26.8	28.7	-	7.63-42.6 pg/mL
β-HCG	<0.2	-	-	<2 UI/L
IGF-1	195	201	-	83.6-259 ng/mL

**Figure 1:**
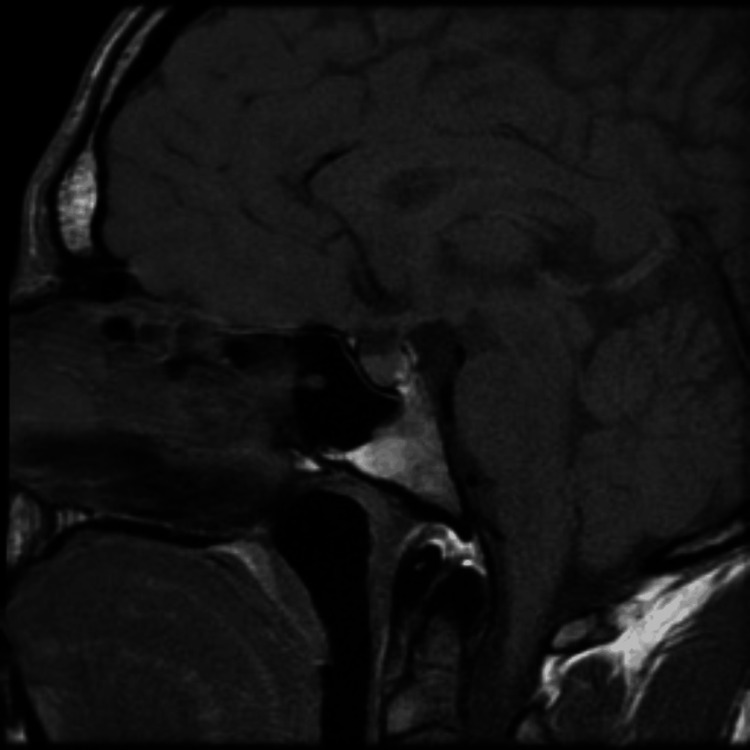
Midline sagittal T1-weighted sellar MRI. Pituitary and sellar area without significant changes. MRI: magnetic resonance imaging

**Figure 2:**
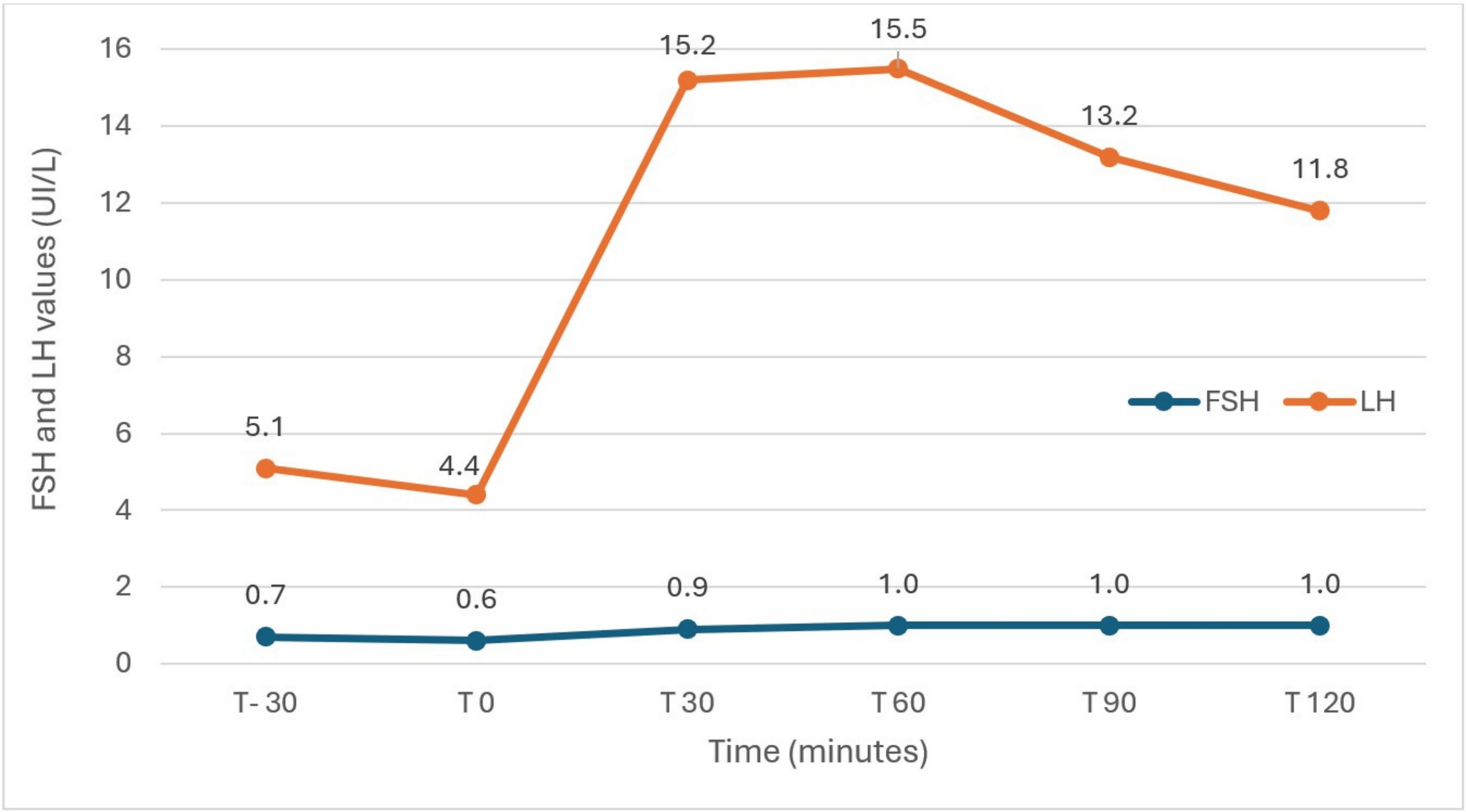
GnRH stimulation test results. GnRH 100 µg was administered as an intravenous bolus; blood was sampled for LH and FSH at -30, 0, 30, 60, 90, and 120 minutes. The test demonstrated an inadequate FSH response (mild increase after 120 minutes) with a normal LH curve (peak increase >3× baseline). GnRH: gonadotropin-releasing hormone; LH: luteinizing hormone; FSH: follicle-stimulating hormone

Testicular ultrasound confirmed normal morphology and volume bilaterally. Follow-up evaluation demonstrated primary autoimmune hypothyroidism, normal total testosterone with adequate LH, and persistent low FSH levels (0.6 UI/L) (obtained through electrochemiluminescence immunoassay (ECLIA)-based kit: cobas®, Roche) (Table 1). Levothyroxine supplementation was initiated. Sellar magnetic resonance imaging (MRI) ruled out any pituitary or pituitary stalk abnormalities (Figure 1). A GnRH stimulation test demonstrated an inadequate FSH response (mild increase after 120 minutes) with a normal LH curve (peak increase >3× baseline) (Figure 2).

Semen analysis was postponed by the patient’s choice. A next-generation sequencing (NGS) gene panel for hypogonadotropic hypogonadism, including *FSHB *gene, was negative. Whole exome sequencing (WES) with screening for copy-number variants (CNVs), fully covering *FSHB *coding regions and flanking introns, was also negative. Due to inconclusive genetic testing, FSH levels were re-measured using an alternative ECLIA-based kit (Atellica®, Siemens), which confirmed the low FSH levels (<0.6 UI/L). At the last follow-up, the patient presented improved gynecomastia after levothyroxine supplementation initiation and decreased opioid-based analgesic use.

## Discussion

To this date, 12 male cases of IFD have been published: five with confirmed *FSHB* mutations, five with no recognized mutations, and two not submitted to genetic testing [3-8]. We describe the first Portuguese case of a man with IFD with no detected FSH-β mutations, even after WES analysis. His gynecomastia was deemed to be multifactorial, considering the untreated hypothyroidism and the possible association with opioids, also supported by his improvement after levothyroxine supplementation and decreased opioid use [9,10]. Additionally, FSH levels remained low even after thyroid function normalization and analgesic use decrease, ruling out untreated hypothyroidism and opioid effects as the most likely causes. In fact, both opioids and hypothyroidism are known to impact hypothalamic GnRH secretion but are unlikely to cause isolated FSH suppression, as both conditions affect the hypothalamic-pituitary-gonadal (HPG) axis in a manner that typically results in both FSH and LH suppression. Primary hypothyroidism may lead to an increase in TRH, which in turn stimulates prolactin secretion that suppresses GnRH, explaining the reduction in both gonadotropins’ output, but not selectively suppressing FSH alone [11,12]. Similarly, chronic opioid use may suppress the HPG axis primarily by inhibiting pulsatile GnRH secretion, which leads to decreased secretion of both LH and FSH. The suppression is described as more pronounced for LH, but FSH is also reduced; isolated FSH suppression is not a recognized opioid effect [13]. Finally, his history of recurrent head trauma could have caused subtle brain injuries that would potentially affect pituitary function, even with a normal MRI, but an isolated FSH gonadal axis impairment would also be highly unlikely.

Regarding fertility, we could not describe to what extent the patient’s sperm count and quality have been affected due to semen analysis postponement, limiting full assessment of clinical IFD. All five reported male cases of IFD without FSH-β gene mutations were diagnosed during infertility evaluation and presented normal pubertal and sexual development and/or moderate testicular hypotrophy with severe oligo/teratospermia [3-6]. Testicular biopsy was registered in only one patient, revealing hypospermatogenesis with decreased spermatids and spermatozoa counts [6]. Recombinant FSH treatment resulted in successful pregnancies in four of these cases [3,5,6].

Our patient differs from all these cases, since biochemical IFD was an incidental finding during gynecomastia evaluation. Due to patient preferences, neither a spermogram nor a testicular biopsy was performed, limiting further understanding of the spermatogenesis process and the clinical impact of his IFD. No mutations that could explain these findings were identified, and we could not exclude the presence of defects in unknown factors involved in FSH regulation or undiscovered mutations that could justify the FSH-deficiency phenotype. FSH structural heterogeneity and β-subunit polymorphisms reflect some of the proposed hypotheses in the literature, although they do not explain his low FSH levels [3]. Nevertheless, there seems to be no clinical differences between patients with and without identified mutations, according to current evidence [3].

There is a paucity of reported pathogenic *FSHB* variants. Only five different single-nucleotide variants (SNVs) are reported in the public archive of human genetic variations ClinVar® (two missense and three truncating), all in exon 3. No homozygous deletions of *FSHB* have been reported. Only heterozygous deletions, substantially larger than *FSHB* and encompassing additional coding genes, are reported in the public archives of human genetic variations Decipher® and ClinVar®; these would not be disease-causing as IFD is AR. Nevertheless, we can confidently exclude a homozygous full deletion in our patient, as the NGS tests would not have any coverage of the gene. It is also unlikely that the patient carries pathogenic intronic variants, as the intronic regions of *FSHB* are small (less than 1 kb) and there is adequate coverage of flanking regions from exons. Other potential causes for this phenotype could be structural variants missed by current genetic testing technologies (e.g., intermediate-size CNVs), variants in trans-regulating genomic regions, or epigenetic changes affecting *FSHB* expression.

## Conclusions

In conclusion, we presented a rare case of biochemical IFD in a male patient incidentally identified during gynecomastia evaluation. Though a definite diagnosis of clinically significant IFD could not be confirmed in the absence of semen analysis, this report highlights the necessity for careful clinical and biochemical assessment in patients with suspected hypogonadism.

*FSHB* mutations represent the most common cause of IFD, although some cases remain genetically unexplained. This underscores the need for further research into regulatory and epigenetic mechanisms involved in FSH production and action, possibly yielding new diagnostic and therapeutic approaches.
